# Estrogen receptor-α regulation of microRNA-590 targets *FAM171A1*—a modifier of breast cancer invasiveness

**DOI:** 10.1038/s41389-018-0113-z

**Published:** 2019-01-09

**Authors:** Rahul Sanawar, Vipin Mohan Dan, Thankayyan R. Santhoshkumar, Rakesh Kumar, M. Radhakrishna Pillai

**Affiliations:** 1 Cancer Research Program, Rajiv Gandhi Centre for Biotechnology, Thiruvananthapuram, Kerala 695014 India; 2 Manipal Academy of Higher Education (MAHE), Manipal, Karnataka 576104 India; 3 Present Address: Jawaharlal Nehru Tropical Botanic Garden and Research Institute, Palode, Thiruvananthapuram, Kerala 695562 India

## Abstract

The pathobiology and aggressiveness of the triple negative breast cancer (TNBC) are influenced by genes that are preferentially expressed in TNBC cells. However, the nature of such genes with the role in invasiveness of TNBC cells is not fully understood. Here, we identified *FAM171A1*, member (A1) of the family with sequence similarity 171, as an overexpressed candidate gene in TNBC cells and tumors as compared to estrogen receptor-alpha (ERα) positive breast cancer. We found that the expression of *FAM171A1* correlates well with the loss of ERα as well as its newly identified target miR590-5p in TNBC but not in ERα-positive cells. In addition, we report that ERα regulates *FAM171A1* expression through a mechanism which involves ERα stimulation of miR590-5p expression via binding to its promoter, and in-turn, miR590-5p suppression of *FAM171A1* expression. Further, we found that the levels of *FAM171A1* correlate well with cancer cell aggressiveness as depletion or overexpression of *FAM171A1* confers reduced or increased ability of TNBC cells to form mammospheres, respectively in accordance with the previous report of increased mammosphere formation potential of metastatic cells. In brief, results presented here have demonstrated that ERα regulation of *FAM171A1* expression via miR590-5p explains the molecular basis of the noticed reduced levels of *FAM171A1* in ER-positive breast cancer cells and that *FAM171A1* is a preferably TNBC- overexpressed gene. Further, the noted loss of ERα–miR590-5p axis may upregulate the expression of *FAM171A1* and consequently, resulting aggressiveness of TNBC cells. These findings suggest that *FAM171A1* might represent a potentially novel therapeutic target for TNBC tumors.

## Introduction

Breast cancer is the most common form of cancer in women^1^. Despite significant advances in our ability to detect and treat breast cancer, it remains a leading cause of death in women with cancer, and the incidence of breast cancer continues to rise in many parts of the world. Among breast cancers, triple-negative breast cancer (TNBC) sub-type is one of the aggressive breast cancers as TNBC cells lack all three currently targetable molecules such as estrogen receptor, progesterone receptor, and HER2.

The family with sequence similarity 171, member A1 protein (FAM171A1) is a glycoprotein composed of 890 amino acids with a molecular weight of 97,854 Da. It is a single-pass type 1 membrane protein, also called “Astroprincin”. Previous work suggests that *FAM171A1* may be associated with chemoresistance of cancer cells^2,3^. Data-mining studies suggest that FAM171A1 interacts with FAM171B, PCDHGB1, TNFRSF17, TMEM, CTDSPL, and NTRK1^4–7^, many of which have been implicated in various cancers^8–13^. Emerging reports suggest that *FAM171A1* might be overexpressed in TNBC tumors^14–16^. However, the nature of the upstream regulation of *FAM171A1* and biological significance of *FAM171A1* in breast cancer remains poorly understood, and this is being addressed in the present study.

miRNAs play a pivotal role in human cancer and act as key regulators during tumor initiation, proliferation, and metastasis and thereby thought to be important as diagnostic, predictive, and prognostic biomarkers. Furthermore, the expression profile of specific miRNAs differs between various subtypes of breast cancers. These subtle differences could be possibly attributed due to the differential signaling cascade initiating from estrogen receptor-alpha (ERα), PGR, and Her2. In this context, miR590-5p is reported to be high in ERα-positive MCF-7 cells as compared to ERα-negative MDA-MB231 cells^17^, while another report found no change in the levels of miR590-5p (also known as OncomiR, anti-anti-OncomiR) between ER+ and ER− breast cancer cells^18^. A previous report shows that the miR590-5p containing genomic region, along with its promoter, resides within the *EIF4H* gene^19^, is an intergenic miRNA^20^. Further, miR590-5p has been shown to be modified by ERα in MCF-7 cells^21,22^, through a poorly understood mechanism.

Here we identify *FAM171A1* as a preferentially expressed gene in basal-type breast tumors, and its levels closely correlate with the aggressiveness of breast cancer cells. Additionally, we explored the role of ERα and its newly identified target here, the miR590-5p, in the regulation of *FAM171A1* expression in ERα+ breast cancer and TNBC cells. We present evidence to demonstrate that overexpression of *FAM171A1* promotes the expression of epithelial-to-mesenchymal (EMT) markers as well as supports the ability of MCF-7 and T47D cancer cells to grow in an anchorage-independent manner and form mammospheres; levels of *FAM171A1* inversely correlate with the status of ERα; and ERα regulates the expression of *FAM171A1* via stimulating the expression of miR590-5p which, in-turn, targets *FAM171A1*. In brief, the present study reveals a new molecular component of breast cancer aggressiveness and raises the possibility of targeting *FAM171A1* in developing novel TNBC-directed future therapeutic approaches.

## Results

### *FAM171A1* overexpression in TNBC breast cancer cell lines and tissues

**Fig. 1:**
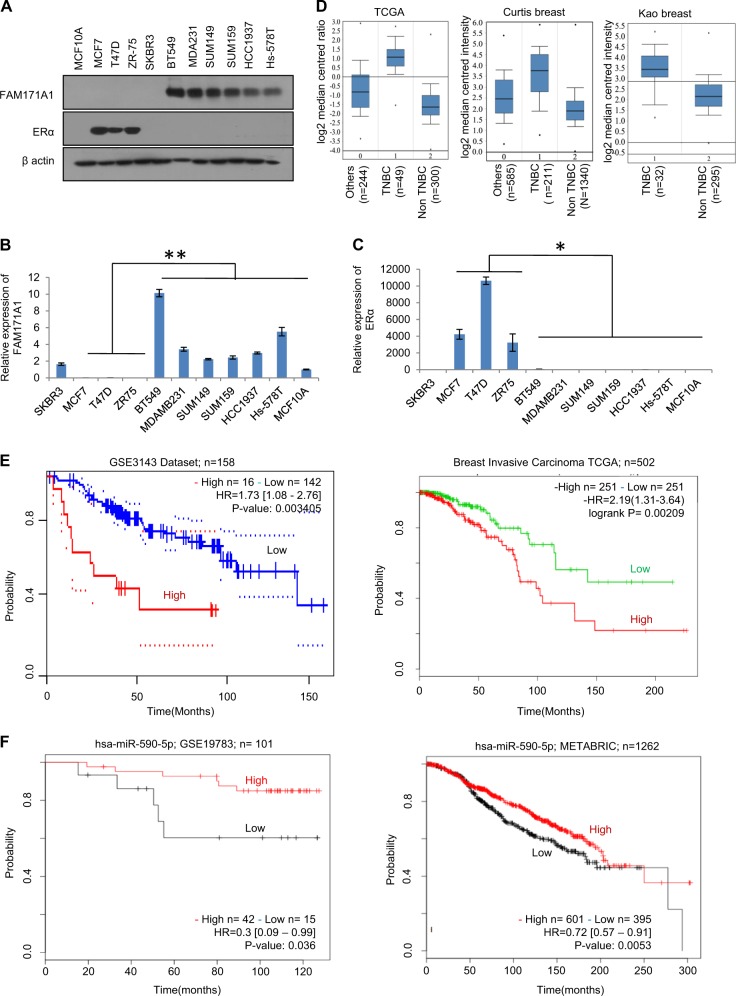
Expression and significance of *FAM171A1* in breast cancer cells and tumors. **a** Western blot analysis for FAM171A1 in a panel of cell lines. **b**, **c** qRT-PCR analysis of FAM171A1 and ERα in breast cancer cell lines. Unpaired two-tailed *t*-test; *p = 0.033 for FAM171A1; **p = 0.002 for ERα. **d** Boxplots showing mRNA expression of FAM171A1 among TNBC and non-TNBC tumor samples in three Oncomine datasets: TCGA, Curtis, and Kao’s breast; datasets ordered by overexpression Gene Rank-top 10%, a threshold at *p* value = 1E−4. **e** Kaplan–Meier survival curve analyses of *FAM171A1* in patients with breast cancer in GSE3143 (*n* = 158) and Breast Invasive Carcinoma TCGA (*n* = 502), datasets stratified based on high (red) and low (blue) in case of GSE3143 dataset and high (red) and low (green) in case of Breast Invasive Carcinoma TCGA; and *p < 0.002, log-rank test. **f** Overall survival curve of breast cancer patients with *hsa-miR590-5p* expression in METABRIC (*n* = 1262) and GSE19783 (*n* = 101), datasets stratified based on high (black) and low (red); log-rank test. All experiments were performed in biological replicates and three technical replicates with similar results

Microarray expression profiles of breast cancer cell lines and breast tumor sub-types such as luminal and basal tumor tissues have been published recently^23^. We analyzed these data sets to identify the nature of unique genes that might be closely overexpressed in TNBC cells and TNBC breast tumors (Supplementary Fig. 1A). This strategy led us to a single gene, known as *FAM171A1*—which is widely expressed in the basal-like breast cancer cell lines and tissue samples. Multiple datasets for post-translational modifications predicted that FAM171A1 might be a glycoprotein, and accordingly, we noticed an expected shift in the mobility of FAM171A1 protein upon exposing MDA-MB-231 cells with tunicamycin, which targets glycosylated proteins (Supplementary Fig. 1B). Next, we screened the status of FAM171A1 in a panel of TNBC and non-TNBC breast cancer cell lines. We found high levels of FAM171A1 in the basal-type TNBC cell lines such as MDA-MB-231, SUM149, SUM159, BT549, HCC1937, and Hs-578T as compared to non-TNBC breast cancer cell lines such as MCF-7, T47D, ZR-75, and SKBR3 as well as normal mammary epithelial cell line, HMEC (Fig. 1a–c, Supplementary 4A, B). Consistent with these observations, levels of *FAM171A1* were higher in TNBC tumors as compared to non-TNBC tumors as analyzed by Oncomine database (Fig. 1d). Kaplan–Meier survival curve analyses of breast cancer patients by the PrognoScan, the Survival Express, and the KM plotter revealed an increased expression of *FAM171A1*, and that its levels correlate well with an overall poor survival of the breast cancer patients (Fig. 1e and Supplementary Fig. 1C). However, Kaplan–Meier survival analyses of miR590-5p (a suspected modifier of *FAM171A1*, this study) show that high expression of miR590-5p may lead to better overall survival rates of breast cancer patients, highlighting its significance in breast cancer biology in physiologically relevant setting (Fig. 1f). In brief, these findings suggest that *FAM171A1* might be preferentially involved in the pathobiology of TNBC tumors.

### ERα downregulates *FAM171A1* expression

**Fig. 2:**
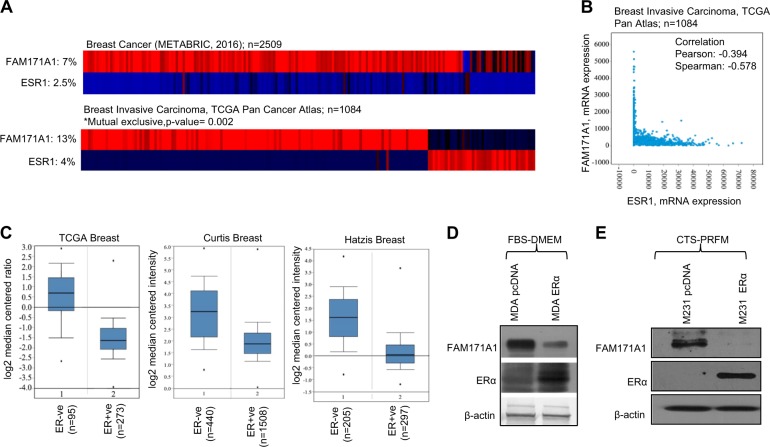
Mutual exclusivity between the expression of *FAM171A1* and *ESR1* (ERα) in breast cancer. **a** Expression of *FAM171A1* and *ESR1* (ERα) in METABRIC (*n* = 2051) and TCGA (*n* = 1084) databases analyzed by cBioPortal tool. **b** Scatter plot analysis of *ESR1* (ERα) and *FAM171A1* mRNA expression using cBioPortal; Pearson correlation: 0.394; Spearman: −0.578. **c** Boxplots showing the mRNA expression of *FAM171A1* in three Oncomine datasets stratified based on ERα positive and ERα negative status; TCGA (*n* = 593), Curtis (*n* = 2136), and Hatzis (*n* = 508); datasets ordered by overexpression Gene Rank; top 10%, a threshold at *p*-value = 1E−4. **d**, **e** Western blot analysis showing the effect of overexpression of ERα on FAM171A1 in MDAMB231 cells cultured in both FBS containing DMEM media and CTS containing phenol-red free DMEM. Experiments (**d**, **e**) were performed three times with similar results

Next, we set to understand the basis of noticed reduced expression of *FAM171A1* in ERα-positive breast cancer cells as compared to ERα-negative breast cancer cells. We tested the hypothesis that ERα might be responsible for the noted reduced expression of *FAM171A1* in ERα-positive breast cancer cells. If this is true, then the loss of ERα in TNBC cells might possibly explain the noticed increased expression of *FAM171A1* in TNBC cells and tumors. To experimentally test this notion, we first analyzed the published breast tumor datasets using cBioPortal tools. We found that indeed, the expression of *FAM171A1* and *ESR1* (ERα) mRNAs are mutually exclusive (Fig. 2a, b). We then performed extensive mining of the Oncomine data resource and found that in general, *FAM171A1* expression negatively correlates with the status of *ESR1* (ERα) (Fig. 2c). As ERα (*ESR1*) expression strongly parallels to the PR (*PGR*; Progesterone receptor), we next tried to assess the correlation between the *ESR1*, *PGR*, and *FAM171A1* in published cell line datasets as well as in other cancer datasets. This analysis revealed that in general, *ESR1* expression exhibits a negative correlation with *FAM171A1* in breast cancer datasets, and a similar trend was also observed for *PGR* in respective cell lines as well as carcinoma databases other than breast cancer datasets (Supplementary Fig. 2A, B, C, and D). These observations raised the possibility of reciprocal regulation of *FAM171A1* by *ESR1* or vice-versa. To address this, we knocked down the levels of endogenous *FAM171A1* in MDA-MB-231 cells and analyzed the expression levels of *ESR1*. We did not observe any effect of *FAM171A1* depletion on the levels of ERα (Supplementary Fig. 1D).

Next, we analyzed the regulation of *FAM171A1* by modulating the status of ERα. We noticed that the ectopic expression of ERα in MDA-MB-231 cells leads to significant repression of *FAM171A1* (Fig. 2d and Supplementary Fig. 1E). To understand the role of weak estrogenic phenol red in modulating the expression of *FAM171A1*, we examined the expression of *FAM171A1* in MDA-MB-231 cells cultured in phenol-red free culture medium supplemented with 5% charcoal-stripped serum and examined the impact of ERα expression upon the status of *FAM171A1*. As suspected, ERα overexpression in the absence of estradiol leads to a reduction in the level of FAM171A1 protein and transcript (Fig. 2e and Supplementary Fig. 1F), suggesting a contribution of experimental culture conditions in residual levels of FAM171A1. Similar results were noticed in SUM149 and SUM159 cell lines (TNBCs) (Supplementary Fig. 4C, D and E). These findings are consistent with the notion that unliganded ERα alone represses multiple gene signatures as supported by the published report^24,25^.

### Regulation of *FAM171A1-*UTR activity by miR590-5p

**Fig. 3:**
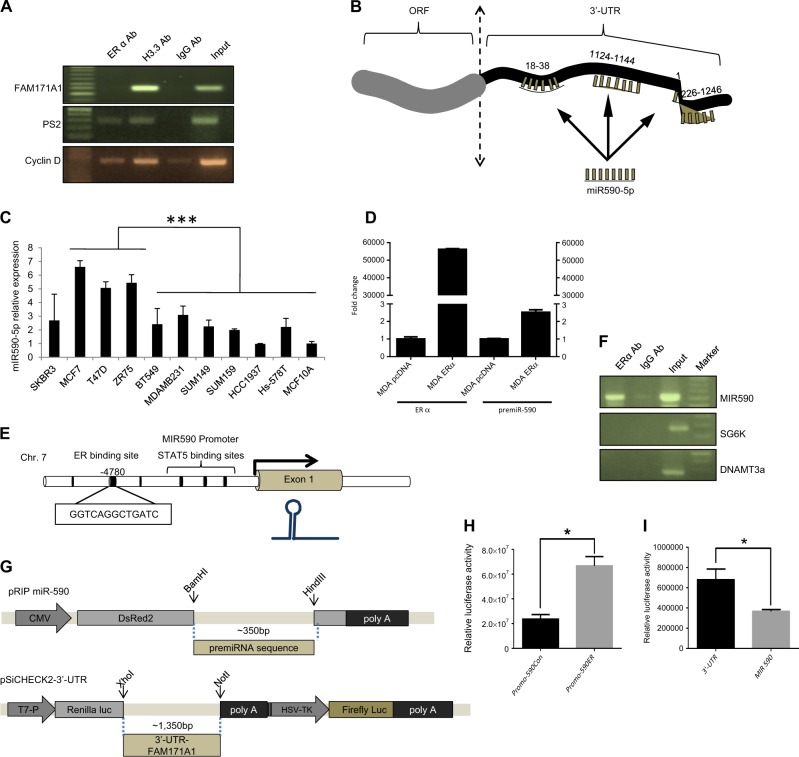
miR590-5p regulation of *FAM171A1* in breast cancer cell lines. **a** Chromatin Immunoprecipitation analysis showing lack of ERα recruitment onto the *FAM171A1* promoter; pS2 and Cyclin D as positive controls. **b** Diagram showing three seed sequences at designated locations within the 3ʹ-UTR of the *FAM171A1* gene. **c** Endogenous expression of miR590-5p in breast cancer cell lines as analyzed by qRT-PCR using TaqMan chemistry; ***p < 0.0003. **d** qRT-PCR showing the induction of preMIR590 expression upon ERα transfection in MDAMB231 cell line. The data were normalized to U6 snRNA. **e** Schematic diagram showing the putative ERα-binding region in the *MIR590* promoter. Exon in light gray color, ERE and STAT5 binding sites in black color. **f** CHIP analysis of ERα binding onto the *MIR590* promoter in MCF-7 and T47D cells. SG6K and DNAMT3A primers were used as negative controls. **g** pSi-CHECK2 and pRIP vector constructs showing the regions for cloning of 3ʹ-UTR region and *MIR590* gene, respectively. **h** Effect of ERα cotransfection upon miR-590 promoter-luc reporter activity; *p-value < 0.05 (Student’s *t*-test). **i** Effect of miR-590-pRIP expression on the 3ʹ-UTR*-*pSi-CHECK2-luc activity; *p-value < 0.05 (Student’s *t*-test). Experiments (**a**, **c**, **f**, **h**, **i**) were performed in three biological replicates and **d** was performed two times each time with three technical replicates

Having shown a repressive role of ERα on *FAM171A1* gene, we next analyzed the mechanism by which ERα regulates the level of *FAM171A1*. Using sequence retrieval tool from EPD database^26^, first we extracted the promoter sequence from −2500 bp to 100 bp relative to transcription start site (TSS) and screened for the predicted transcription factor binding motifs within the *FAM171A1* promoter using ALGGEN-PROMO database while keeping dissimilarity margin less or equal than 0%. We found that *FAM171A1* promoter contains one ERE consensus sequence (GGTCAGACTGACT) at −1261 bp. In addition, we also used the JASPAR database, setting up the *p*-value to 0.0001, and found only two ERE sites at the locations −15 bp and −1525 bp. However, ChIP experiments using anti-ERα-Ab failed to exhibit any recruitment of ERα onto the promoter of *FAM171A1* as opposed to the recruitment of ERα onto the promoters of PS2 and Cyclin D1, two ERα responsible genes (Fig. 3a).

Since we observed repression of *FAM171A1* mRNA upon ERα transfection in MDA-MB-231 cells both in the case of activated and unliganded ERα (presumably, due to the ligand-independent action of ERα), we next searched for another possible mechanism of *FAM171A1* regulation by ERα. We examined for the status of specific miRNAs in the context of ERα-positive cancer cells. We identified miR590-5p as a candidate gene with three seed sequences within a single 3ʹ-UTR sequence of *FAM171A1* using “microrna.org” database (Fig. 3b). miR590-5p has been shown to be well expressed in MCF-7 cells compared to MDA-MB-231 cells^17,21^. To validate these results, we determined the status of endogenous miR590-5p in breast cancer cells. Our result shows that the levels of miR590-5p are elevated in ERα-positive breast cancer cells than in ERα-negative breast cancer cells (Fig. 3c).

### ERα regulation of *FAM171A1* via *MIR590* expression

Next, we examined the effect of ERα on the levels of endogenous miR590-5p in MDA-MB-231 cells and found that ERα overexpression upregulates the level of pre-miR590 in breast cancer cells (Fig. 3d). To investigate whether miR590-5p targets the 3ʹ-UTR region of *FAM171A1* upon modulation of ERα. We used miRStart database and noticed that miR590-5p promoter contains a single ERα binding site at the location −1050 bp upstream of TSS (−4780 from the precursor miR590) (Fig. 3e). We found that ERα gets recruited onto the miR590-5p promoter in ChIP assays, as opposed to the promoters of SG6K and DNAMT3a—two ERα unresponsive genes (Fig. 3f). To examine a direct role of ERα in the expression of miR590-5p, we cloned miR590-5p promoter and demonstrated that ERα stimulates miR590-5p transcription (Fig. 3h).

To establish a direct role of miR590-5p in the regulation of *FAM171A1* expression, we cloned the 3ʹ-UTR region of *FAM171A1* into a psi-CHECK2 vector while miR590-5p was cloned into a pRIP vector (Fig. 3g). We found that miR590-5p binds and suppresses the transcription from *FAM171A1*–3ʹ-UTR reporter system (Fig. 3i). Together these findings suggest that ERα stimulates miR590-5p, which, in-turn, represses *FAM171A1* expression, and that the noticed increased expression of *FAM171A1* might be associated with low levels of miR590-5p in ERα-negative TNBC breast cancer cells.

### *FAM171A1* regulation of TNBC biology

**Fig. 4:**
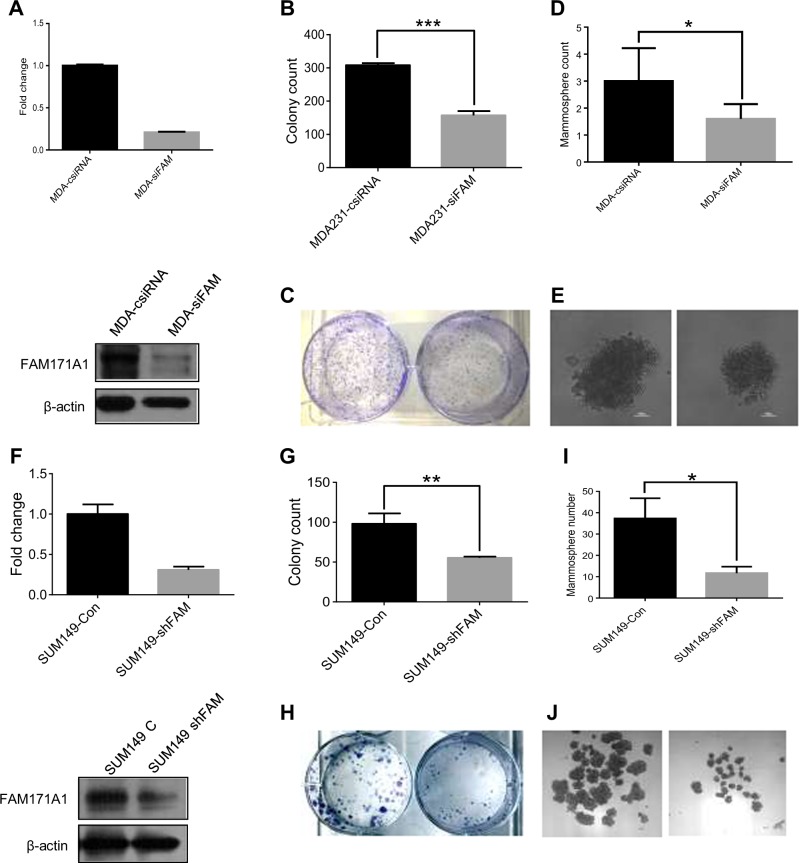
Effect of *FAM171A1* depletion on the growth of breast cancer cells. **a** qRT-PCR and Western blot analyses of *FAM171A1* mRNA and protein upon silencing in MDA-MB-231. **b**, **c** Anchorage-independent colony formation assay showing a decreased proliferation of MDA-MB231 cells upon *FAM171A1* knockdown. **d**, **e** Representative image of the tumorspheres formed by MDA-MB-231 vs silenced MDA-MB-231 cells; scale bar: 100 µm; quantification on the top. **f** Status of *FAM171A1* mRNA and protein in SUM149-vector and SUM149-sh*FAM171A1* stable cell line. **g**, **h** Anchorage-independent colony formation assay showing a decreased proliferation of SUM149 cells upon stable knockdown of *FAM171A1*; quantification on the top. **i**, **j** Representative image of the tumorspheres formed by SUM149 vector vs. SUM149-sh*FAM171A1* stable cells; scale bar: 25 µm, quantification on top; ***p-value < 0.0003; **p-value < 0.005; *p-value < 0.05; unpaired two-tailed *t*-test. All experiments were performed three times and with three technical replicates where needed

To understand the functional significance of the noticed increased expression of *FAM171A1* in breast cancer biology, we evaluated the effect of depleting endogenous *FAM171A1* in MDA-MB-231 cells on its invasiveness, anchorage-independent growth, and ability to form mammospheres. We found that depletion of *FAM171A1* leads to a reduced invasiveness of MDA-MB-231 cells as supported by reduced expression of EMT markers such Snail, Slug, E-Cadherin as well as decreased invasion as assessed by invasion assay in MDA-MB-231 cells suggesting a role of *FAM171A1* in supporting cell invasiveness of TNBC cell (Supplementary Fig. 3A and 3B). In addition, *FAM171A1* knockdown in MDA-MB-231 cells is also accompanied by a reduction in the colony-forming ability of MDA-MB-231 cells (Fig. 4a, b, and c). Interestingly, we also noticed that *FAM171A1* knockdown in MDA-MB-231 leads to a reduced number and size of mammospheres (Fig. 4d and e) as well as reduced expression of stem cell markers such as Oct4 and Sox2 (Supplementary Fig. 3C). Similar to MDA-MB-231 cells, we also found that stable knockdown of *FAM171A1* in SUM149 TNBC cells is also accompanied by reduction in the levels of EMT markers such as Snail, Slug, and Zeb1 assessed by Western immunoblotting (Supplementary Fig. 3D) and reduced ability of cells to form anchorage-independent colonies as well as mammospheres (Fig. 4f, g, h, i, and j). Further, to evaluate the association of *FAM171A1* with stemness, we utilized Gene Set Enrichment Analysis (GSEA) of GSE36693 dataset in order to see any possible overlap between profiles with high expression of *FAM171A1* and stem cell gene signatures. Analysis revealed that high expression of *FAM171A1* correlates with upregulated stem cell gene signatures and therefore, may be involved in the regulation of cancer stem cell functions (Supplementary Fig. 5A). Additionally we also observed activated gene signatures for TGFβ/Smad and Wnt/β-catenin pathways^27,28^ suggesting, these stem cell-associated pathways could possibly be linked with *FAM171A1* (Supplementary Fig. 5B).

**Fig. 5:**
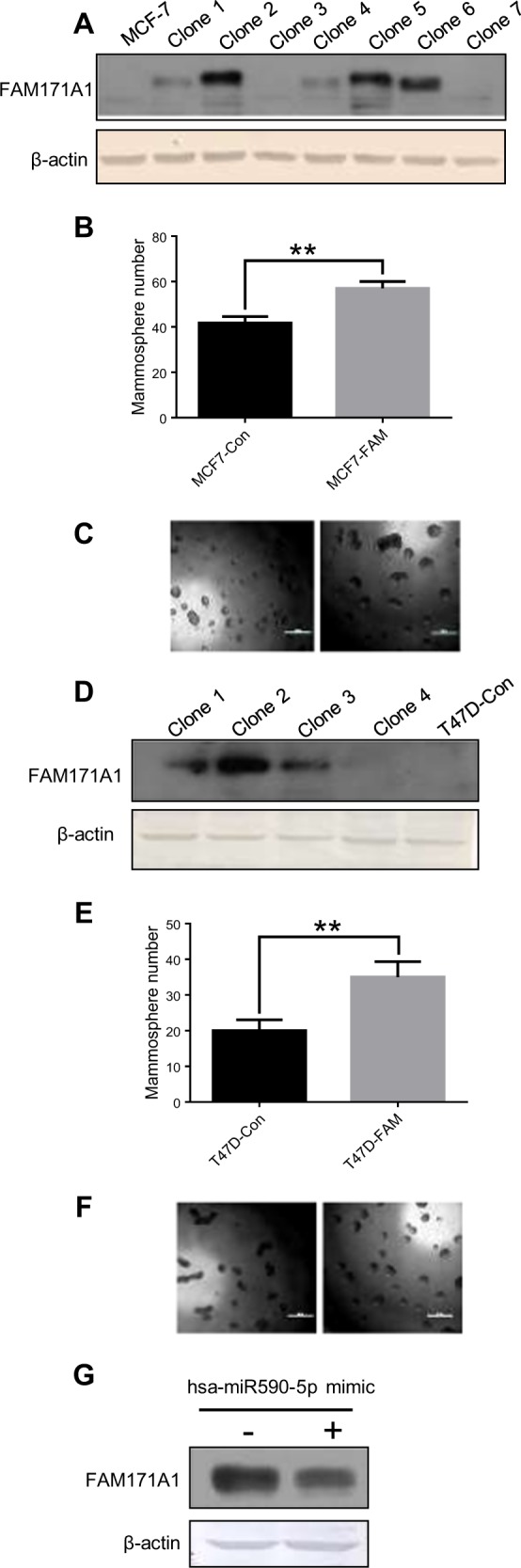
Effect of *FAM171A1* overexpression on the growth of breast cancer cells. **a** Western blot analysis of FAM171A1 expression in MCF-7 stably overexpressing *FAM171A1*. **b**, **c** Representative images of tumorspheres formed by the indicated cells (below); scale bar: 50 μm, quantification on top; **p-value = <0.005, unpaired two-tailed *t*-test. **d** Western blot analysis of FAM171A1 in T47D stably expressing *FAM171A1*. **e**, **f** Representative images of tumorspheres formed by the indicated cells (below); scale bar: 50 μm, quantification on top; **p-value < 0.005, unpaired two-tailed *t*-test. **g** Reduction in the levels of *FAM171A1* expression upon miR-590-5p mimic treatment in MDA-MB-231 cells. All experiments were performed three times with similar results

To further examine the role of *FAM171A1* in supporting the stemness of breast cancer cells, we next generated stable clones from MCF-7 and T47D breast cancer cells which otherwise express no or low levels of *FAM171A1* (Fig. 5a, d). We found that *FAM171A1* overexpression in MCF-7 and T47D cells leads to increased mammosphere-forming capability (Fig. 5b, c, e, f and Supplementary Fig. 3E) as well as EMT markers such as Snail, Slug, Zeb1, and N-Cad in case of MCF-7/*FAM171A1* cells whereas Snail, Slug, Zeb1, Vimentin, and Claudin-1 in case of T47D/*FAM171A1* stable cells (Supplementary Fig. 3F, 3G, and 3H). These differences could be attributed to the differences in the cell line’s gene expressions. Ectopic expression of *FAM171A1* also increased stem cell markers such as Oct4, Sox2, and Nanog as observed in MCF-7/*FAM171A1* as well as T47D/*FAM171A1* stable clones (Supplementary Fig. 3I and 3J) as compared to the control cells. These results suggest that one of the roles of *FAM171A1* in breast cancer cells might be to support their stemness, and in turn, aggressiveness^29^.

**Fig. 6:**
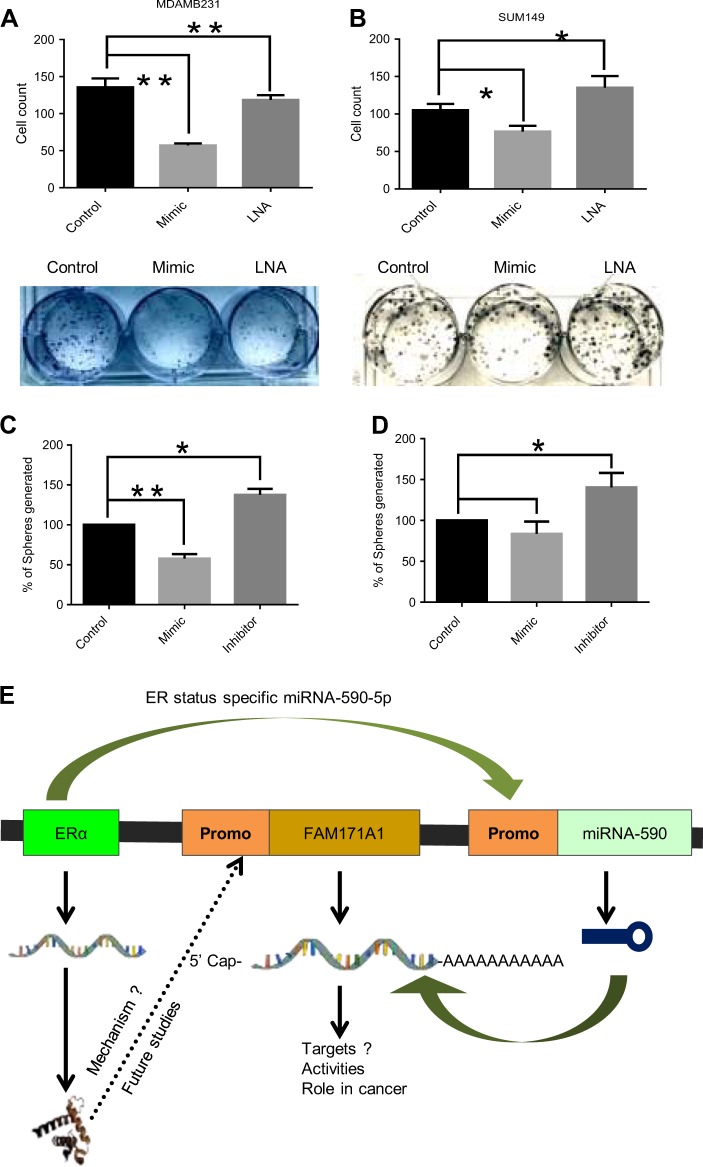
Growth regulation of breast cancer cells by miR590-5p. **a**, **b** Effect of miR590-5p mimic and LNA treatments on the growth of MDA-MB-231 and SUM149 as assessed by colony formation assay; quantification on top. **c**, **d** Quantification of the tumorspheres formed upon miR-590-5p mimic and LNA treatments in MDA-MB-231 and SUM149 cells; *p-value < 0.05, one-way ANOVA; **p-value < 0.005. **e** Working model showing the summary of the regulation of the ERα–miR590-5p–*FAM171A1* axis in breast cancer cells. Experiments were performed in two biological replicates each with two technical replicates with similar results

We next examined the effect of modulating the endogenous levels of *FAM171A1* with exogenously supplied synthetic miR590-5p mimic/LNA (locked nucleic acid) on the ability of cells to form mammosphere. Result in Fig. 5g illustrates that miR590-5p mimic treatment in SUM149 cells reduced the expression of the *FAM171A1* protein (Fig. 5g). Finally, we validated that miR590-5p mimic inhibited the ability of MDA-MB-231 and SUM149 cells to form anchorage-independent colonies and mammosphere, whereas miR590-5p LNA closely increases the ability to form anchorage-independent colonies and mammosphere (Fig. 6a, b, c, and d). Altogether, these findings support the notion that miR590-5p is an endogenous regulator of *FAM171A1* expression and that increased expression of *FAM171A1* in TNBC cells contributes to invasive phenotypes.

## Discussion

In summary here, we demonstrate *FAM171A1* as a candidate gene overexpressed in TNBC cell lines and TNBC tumors as compared to ERα-positive breast cancer cells. The levels of *FAM171A1* are albeit in case of immortalized mammary epithelial cells when compared with ERα-positive or ERα-negative cell lines. High expression of *FAM171A1* is associated with overall poor survival of the breast cancer patients, in contrast to miR590-5p, whose elevated expression may lead to better overall survival rates as assessed by the KM plots suggesting an inverse correlation between clinical significance of these two genes in the survival of breast cancer patients. In silico and data-mining analyses suggest that FAM171A1 has been predicted to interact with FAM171B, PCDHGB1, TNFRSF17, TMEM, CTDSPL, and NTRK1, many of which have been already implicated in various cancers suggesting most likely its possible role in the tumorigenesis. ERα, as well as its target *PGR* (PR), is an established biomarker in breast cancer patients. However, we observed a strong correlation between *FAM171A1* and *ESR1* (ERα) but not with *PGR*. This provided the basis of exploring the underlying relationship between ERα and *FAM171A1* utilizing bioinformatics datasets, such as Oncomine, TCGA, cBioPortal, and in previously published raw microarray data. Here for the first time, we provide the molecular basis of the noted loss of *FAM171A1* that is highly expressed in ERα-deficient breast cancer cells.

During the course of this study, we identified miR590-5p as an ERα target, the expression of which is elevated in ERα-positive, non-TNBC cells such as MCF-7, T47D, and ZR-75 cells when compared to ERα-negative (TNBC) cell lines such as MCF10A, BT549, MDA-MB-231, SUM149, SUM159, HCC1937, and Hs-578T cells. Further, using the ChIP coupled with Luciferase assays, we found that ERα induces the expression of miR590-5p by binding to its promoter and positively regulates its expression.

The underlying mechanism of the noticed upregulation of *FAM171A1* in TNBC cells includes reduced levels of miR590-5p in TNBC cells, which otherwise targets *FAM171A1* in ERα-positive breast cancer cells, and thus the loss of miR590-5p regulates *FAM171A1* in TNBC cells. Mechanistically, we show that miR590-5p is an ERα-responsive gene and that ERα stimulates the expression of miR590-5p, which, in turn, downregulates *FAM171A1* in ERα-positive breast cancer cells. These findings suggest that absence of ERα, as well as its target miR590-5p in TNBC cells, contribute, at least, in part, to the observed increased expression of *FAM171A1* in breast cancer cells. Functional studies showed that elevated expression of *FAM171A1* in breast cancer cells contributes to the invasiveness and stemness of breast cancer cells.

GSEA reveals an overlap between the high level of *FAM171A1*, upregulated stem cell gene signatures as well as activated gene signatures for TGFβ/Smad and Wnt/β-catenin pathways, suggesting a possible involvement of FAM171A1 in the modulation of cancer stem cells. We propose that the noted absence of ERα and its target miR590-5p in TNBC cell lines might be one of the contributing factor for driving the observed elevated endogenous expression of FAM171A1. Future studies are needed to understand the nature of signaling pathways under the influence of *FAM171A1* and their targets responsible for the observed biological functions of FAM171A1. In brief, results presented here suggest that the *FAM171A1* is an important TNBC-specific gene as its level profoundly modulates the aggressiveness of breast cancer cells. To the best of our knowledge, this is the first report showing the mutual exclusivity between the levels of *FAM171A1* and ERα, and underlying mechanism, at least, in part, wherein ERα influences the expression of *FAM171A1* via miR590-5p.

## Materials and methods

### Materials

Breast cancer cell lines MCF-7, T47D, MDA-MB-231, SKBR3, BT549, ZR-75, and Hs-578T were maintained in Dulbecco’s modified Eagle’s medium (DMEM) with 10% fetal bovine serum (FBS), 100 U/ml penicillin, and 100 µg/ml streptomycin. HCC1937 (breast cancer cell line) was cultured and maintained in RPMI medium whereas MCF10A, SUM 149, and SUM 159 (breast cancer cell lines) were cultured and maintained in Mammary Epithelial Cell Growth medium (MEGM^TM^ from Lonza) with fibroblast growth factor, heparin, bovine pituitary extract, hEGF, insulin, hydrocortisone, and gentamicin/amphotericin. Total RNA was isolated from all the cell lines using TRIzol reagent (Invitrogen), and MIRVANA kit was used for the isolation of miRNAs according to the manufacturer’s instructions. TaqMan® MicroRNA Assays for hsa-miR-590-5p, hsa-miR-590–3p*, mir*Vana® miRNA mimic, *mir*Vana® miRNA inhibitor, and *mir*Vana® miRNA negative control were used from Invitrogen company. Simple ChIP® Kit (Cat #9003) was obtained from CST. Antibodies against FAM171A1 (GTX120226), HER2 (CST 2908), ERα (sc-8005), HSC70 (sc-7298), and β-actin(C4) (sc-47778) were purchased from respective companies as indicated by the alphabets on the catalog numbers. Snail (C15D3), Slug (C19G7), E-cadherin (24E10), N-cadherin (D4R1H), Vimentin (D21H3), Zeb1 (D80D3), and Claudin-1 (D5H1D) antibodies were used from Cell Signaling Technology. *FAM171A1* siRNA (sc-90617) and ERα siRNA (sc-29305) were ordered from Santa Cruz Biotechnology (Santa Cruz, CA, USA). pEGFP-C1-ERα (28230) plasmid was obtained from Addgene. *FAM171A1* shRNA (human) was procured from Sigma (TRCN0000264229). Primers used in the study are enlisted in Table 1.

**Table 1:** List of primers used for the study

Gene name	Primers used
*FAM171A1*	Forward 5′-CCTCGCGTTCATTTCCAGAG-3′ Reverse 5′-GGCGTTCCATTACTGCTCAG-3′
*ESR1* (ERα)	Forward 5′-CCACCAACCAGTGCACCATT-3′ Reverse 5′-GGTCTTTTCGTATCCCACCTTTC-3′
*HER2*	Forward 5′-AACTGCACCCACTCCTGTGT-3′ Reverse 5′-TGATGAGGATCCCAAAGACC-3′
*GAPDH*	Forward 5′-CACCAGGGCTGCTTTTAACTCTGGTA-3′ Reverse 5′-CCTTGACGGTGCCATGGAATTTGC-3′
*FAM*(ChIP)-ERα1	Forward 5′-CCCAGCCAAGTCTGGAGGT-3′ Reverse 5′-GGGCACAACTGAGCCTAGAAG-3′
*FAM*(ChIP)-ERα2	Forward 5′-GAGGGTCTTCTTTGCTGTGC-3′ Reverse 5′-ACCAGGTGCCATCCTAATGT-3′
*PS2*(ChIP)	Forward 5′-TTAGCTTAGGCCTAGACGGAATGG-3′ Reverse 5′-GACGACATGTGGTGAGGTCATCTT-3′
*CYCLIN-D*(ChIP)	Forward 5′-CATTCAGAGGTGTGTTTCTCCC-3′ Reverse 5′-CTCAGCGACTGCATCTTCTTTC-3′
*FAM*-CHIP-ERα3	Forward 5′-GAGGGTCTTCTTTGCTGTGC-3′ Reverse 5′-TCAGGAGGAATGGTACTTGGA-3′
*Snail*	Forward 5′-AATCGGAAGCCTAACTACAGCGAG-3′ Reverse 5′-CCTTGGCCTCAGAGAGCTGG-3′
*Slug*	Forward 5′-AGATGCATATTCGGACCCAC-3′ Reverse 5′-CCTCATGTTTGTGCAGGAGA-3′
*Vimentin*	Forward 5′-GACAATGCGTCTCTGGCACGTCTT-3′ Reverse 5′-TCCTCCGCCTCCTGCAGGTTCTT-3′
*E-cadherin*	Forward 5′-GAAGGTGACAGAGCCTCTGGAT-3′ Reverse 5′-GATCGGTTACCGTGATCAAAATC-3′
*N-cadherin*	Forward 5′-ACAGTGGCCACCTACAAAGG-3′ Reverse 5′-CCGAGATGGGGTTGATAATG-3′
*ZEB1*	Forward 5′-GGCAGAGAATGAGGGAGAAG-3′ Reverse 5′-CTTCAGACACTTGCTCACTACTC-3′
*Pre-miR-590*	Forward 5′-GGCTATCCTCTCAGAGTGACATTT-3′ Reverse 5′-GCTTTATCAGGTTATGTTGCATGGT-3′
*miR-590*-*5p*	Forward 5′-AAAGGATCCGTGGGTGAGTATGGGAGGGA-3′ Reverse 5′-AAAAAGCTTGCCAAGCTAAGCCAAGGGTA-3′
*miR-590* promoter	Forward 5′-CTTTATGCTGACCTGCTTG-3′ Reverse 5′-TCTCCTTGTTGCCTGATT-3′
*miR-590*(ChIP)	Forward 5′-TCAAGCGATTCTCCTGC-3′ Reverse 5′-AAGCGTCGTTTACTCTGC-3′
*SG6K*(ChIP)	Forward 5′-TGCAAGGAGAACATTGAACACAA-3′ Reverse 5′-CCGGCGTGCCACAGA-3′
*DNAMT3A*(ChIP)	Forward 5′-AGGAACCTAGAGCCCTGAGC-3′ Reverse 5′-GTGAGTTCCCCGTACCTTGA-3′
*FAM* −3′-UTR	Forward 5′-AAACTCGAGAGACCCACCTGACTGTGGAATATAA-3′ Reverse 5′-AAAGCGGCCCCTTTTCTTCAGAGTTTATTTTGAA-3′
*U6* snRNA	Forward 5′-CTCGCTTCGGCAGCACA-3′ Reverse 5′-AACGCTTCACGAATTTGCGT-3′

### Western blot analysis

Cells were harvested and lysed in 50–100 µl lysis buffer. Lysates were clarified by centrifugation at 14,000 rpm for 15 min. A total of 50 μg protein was denatured, processed, and loaded for SDS-PAGE, which was performed using 8% gels. The proteins were electrophoretically transferred to PVDF membranes (Amersham^TM^ Hybond^TM^ P 0.23 µm PVDF). The blots were then blocked with 3% BSA in Tris-buffered saline (TBS, pH 7.6) for 1 h and then incubated with primary antibodies in 3% BSA in TBST overnight. After washing with TBST (TBS with 0.1% Tween-20), the membranes were incubated with HRP conjugated secondary antibodies for 1 h at room temperature and were visualized by enhanced chemiluminescence (MILLIPORE Immobilon^TM^ Western HRP Substrate Luminol Reagent) and developed on X-ray film (Carestream, Colorado, USA).

### Knockdown/overexpression studies

MDA-MB-231 cells were seeded in 6-well plates 1 day before transfection to reach 70% confluency by the next day. For overexpression studies, cells were transfected with 1 µg plasmids, and for knockdown studies, 120 pmol of *FAM171A1* siRNA was standardized and used for the efficient knockdown. Plasmid transfections and shRNA transfection was done using Lipofectamine® LTX and Plus^TM^ Reagent (Invitrogen, Carlsbad, CA, USA) and Lipofectamine 3000 reagent whereas siRNA transfection was done using Lipofectamine RNAi Max (Invitrogen, Carlsbad, CA, USA) according to manufacturer’s protocol.

### Quantitative reverse transcription-PCR

Total cellular RNA was extracted using TRIzol, and cDNA was synthesized using High Capacity cDNA Reverse Transcription Kit (Applied Biosystems, USA). cDNAs were used for quantitative reverse transcription-PCR (qRT-PCR) analysis using SYBR-Green Master PCR mix (Roche) on a Step One Plus Real-Time PCR system (Applied Biosystems, USA). GAPDH was used as a normalizing control in the case of gene expression studies whereas RNU48 was used as an endogenous control in case of miRNAs expression study. For individual quantitation of miRNA, RNA isolation was performed using mirVana™ miRNA Isolation Kit (Cat. No. AM1560; Thermo Fisher Scientific). About 50 ng of total RNA was converted to cDNA using TaqMan miRNA Reverse Transcription kit (ABI) according to the manufacturer’s guidelines, which was then analyzed using TaqMan probes *miR-590-5p, miR-590-3p*, and *RNU44/48* obtained from Invitrogen.

### Bioinformatics studies

To examine the binding of ERα to the *FAM171A1* promoter, we analyzed 2.5-kb promoter sequence upstream of TSS for the predicted transcription factor binding sites using the transcription factor binding tools—JASPAR, ALGGEN PROMO, and EPD databases. Promoter motifs such as TATA box, CCAAT box, GC box as well as initiator motif were analyzed by Eukaryotic Promoter Database. *FAM171A1* gene is transcribed from the minus strand. Since no validated transcription factors are reported for *FAM171A1* gene so far, we tried to predict the transcription factors bound to its promoter based on the least dissimilarity score of “0” in case of ALGGEN PROMO whereas *p*-value 0.0001 for EPD and JASPAR. Conventionally, we set out to find the presence of ERE (ER response element, GGTCAnnnTGACC) sequence in the promoter region. We noticed a perfect ERE consensus sequence in the promoter of *FAM171A1* within 2 kb region. This raised the possibility that ERα could bind to the *FAM171A1* promoter either directly or through a co-regulatory complex. For the ERα binding site within the *MIR590* promoter, we analyzed ~2.5-kb upstream sequence from TSS for *MIR590* gene.

### Chromatin immunoprecipitation

MCF-7 and T47D cells were grown in three 100 mm culture dishes. After 80% confluency was reached, the cells were fixed with formaldehyde and CHIP protocol was performed using SimpleChiP^TM^ Enzymatic Chromatin IP Kit (Agarose Beads) from Cell Signaling Technology according to their protocol. For sonication, 14 cycles were standardized for the required fragment sizes with 30 s ON and 30 s OFF. The ChIP products were analyzed by PCR for both *FAM171A1* as well as miRNA-590 promoter’s binding using the primers listed above in the text.

### Colony formation assay

One-thousand cells were seeded in each well of 6-well plates for MDA-MB-231 and 500 for SUM149. The plates were kept back in the CO_2_ incubator for nearly about 10–12 days. The media were changed every 3 days. On the last day, the media were aspirated, and the wells were washed with 1× PBS very carefully. Colonies were fixed with 10% neutral formalin (1 ml formalin for each well of 6-well plate) for nearly half an hour. Then the wells were again rinsed with 1× PBS carefully. Colonies were then stained with 1 ml of 0.5% crystal violet stain, incubated for 30 min, and wells were destained under running tap water very carefully.

### Mammosphere formation assay

Cells were grown in T-25 flask up to 70–80% confluency, trypsinized and resuspended in MEBM media containing 10% FBS and reseeded in 96-well low attachment surface plates containing 200 cells in each well having 200 µl of MEBM media. Plates were then incubated in the CO_2_ incubator for 10–12 days for the formation of spheres. After 10–12 days, both the number and the size of the spheres were observed and counted.

### Invasion assay

The protocol followed was according to manufacturer’s guidelines. Briefly, a plate was taken out from −20 °C and kept at room temperature for nearly 10–15 min; 500 µl of warm (37 °C) DMEM was added to each apical chamber and kept for 2 h in a 37 °C incubator with 5% CO_2_. After rehydration, the medium was carefully removed from each apical chamber without disturbing the layer of BD Matrigel™ Matrix on the membrane. Cell suspensions were made in serum-free DMEM and 2 × 10^4^ cells were seeded on to the top of each apical chamber. Finally, the total volume of the cell suspensions was made up to 500 µl by adding additional serum-free DMEM to the apical chamber if required. Lower chambers were filled with 750 µl of 5% FBS serum containing DMEM as a chemo-attractant. BD BioCoat™ Tumor Invasion System and the uncoated BD Falcon FluoroBlok™ 24-Multiwell Insert System were then incubated for 20 h at 37 °C, 5% CO_2_. Following incubation, the medium was removed from apical as well as the lower chamber, replaced with Hoechst dye (2 µg/ml) containing phenol red-free DMEM in the lower chamber and incubated for 15 min. Then, the dye-containing medium was aspirated out and replaced with 1× PBS for washing the lower side of the apical chamber carefully just by dipping. Invaded cells were imaged and counted by nuclear stain.

### miR-590 promoter luciferase assay

MDA-MB-231 cells were transfected with 200 ng of pGL3 vector cloned with the *miRNA-590* promoter or pGL3 control; 50 ng of Renilla luciferase vector was transfected, serving as a transfection control. The cells were co-transfected with pcDNA3.1 and ERα vectors where necessary. After 48 h, the cells were lysed using Glo Lysis Buffer (Promega), and the lysate was clarified by centrifuging at 14,000 rpm for 15 min. Firefly and Renilla luciferase activities were measured using RenillaGlo and SteadyGlo reagents (Steady-Glo-Luciferase Assay System and *Renilla*-Glo-Luciferase Assay System from Promega) according to manufacturer's guidelines. Firefly readings were normalized to renilla luciferase readings to generate relative luciferase activity.

### 3ʹ-UTR luciferase assay

MCF-7 cells were seeded into 24-well plates to 80% confluency. Next day, the cells were transfected using Lipofectamine LTX plus with 200 ng psiCHECK2 vector encoding the entire 3′-UTR region of *FAM171A1* gene fused downstream of the renilla luciferase gene and the firefly luciferase gene as a reporter along with 200 ng pRIP plasmid constructs encoding miR-590 or pRIP control plasmid vector where required and the firefly luciferase gene as a reporter. After 48 h incubation, the cells were assayed with the Dual-Luciferase® Reporter Assay System (Promega) to measure the renilla luciferase and firefly luciferase activity, which served as a transfection control. In brief, the 24-well plate was taken out, and the cells were washed once with 1× PBS. For cell lysis, 100 µl of 1× Passive Lysis Buffer was added to each well and incubated for 15 min over a shaker at maximum speed for efficient lysis. Cells were then scraped/pipetted up–down. Lysed cells were collected in microfuge tubes and centrifuged for 15 min at 14,000 rpm. The supernatant was collected (~100 µl) and stored at −80 °C. Luciferase readings were taken using a Luminometer (TD20/20, Promega) system according to manufacturer’s guidelines.

### miR590-5p LNA/mimic experiment

About 2.5 × 10^5^ cells were seeded in 6-well plates. For transfection, 30 nM miR590-5p mimic, as well as the inhibitor, were used. RNAimax from Invitrogen was used for transfection according to the manufacturer’s protocol. Post 48 h, the cells were trypsinized and seeded for colony formation as well as sphere formation assay.

### Statistics

Where necessary, statistical data were analyzed using GraphPad Prism to generate SE values and to determine the level of significance using the Student’s *t*-test (one-tailed or two-tailed, as appropriate) and one-way ANOVA; *p-value < 0.05; **p-value 0.005, ***p-value 0.0001 were considered to indicate significance. Data are reported as mean ± SEM.

## Supplementary information

SUPPLEMENTARY INFORMATION

REBUTTAL LETTER
