# Supplementary material for: Estrogen receptor-α regulation of microRNA-590 targets FAM171A1—a modifier of breast cancer invasiveness
Source: Oncogenesis. 2019 Jan 9;8(1):5. doi: 10.1038/s41389-018-0113-z (PMC6328622; doi:10.1038/s41389-018-0113-z)
Supplement: Supplementary file 1 — SUPPLEMENTARY INFORMATION [file 41389_2018_113_MOESM1_ESM.pdf]

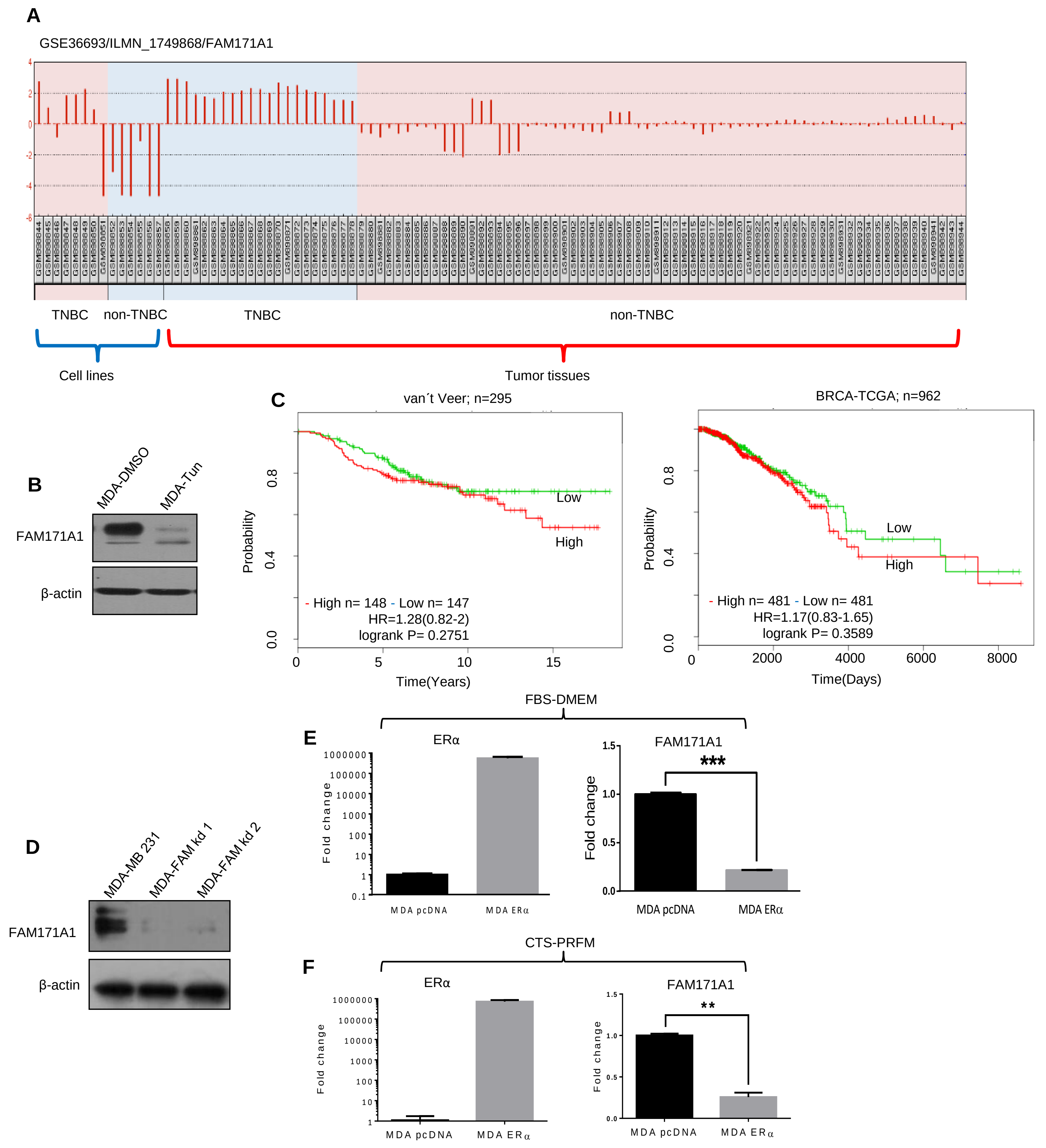

Supplementary Figure 1

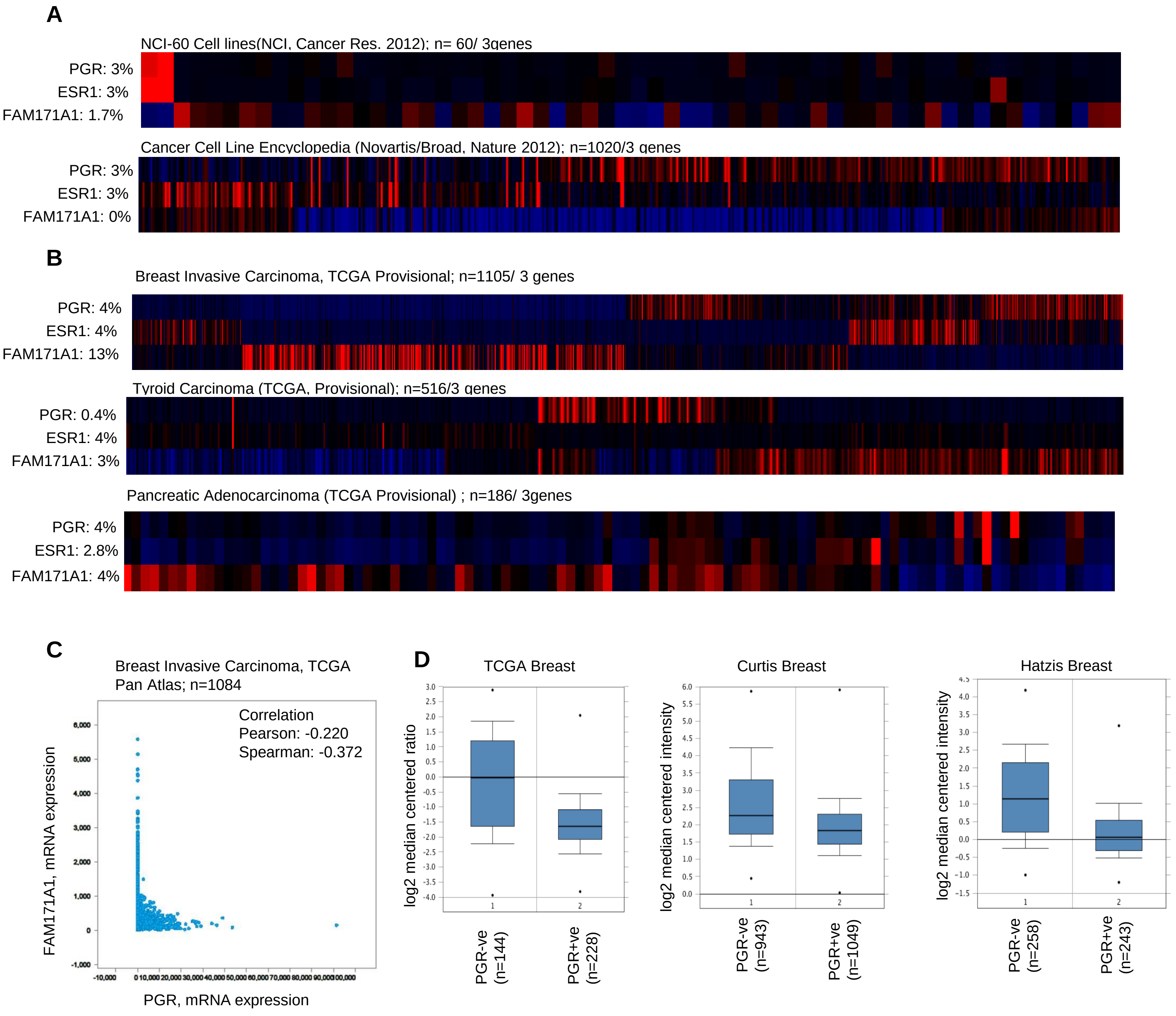

Supplementary Figure 2

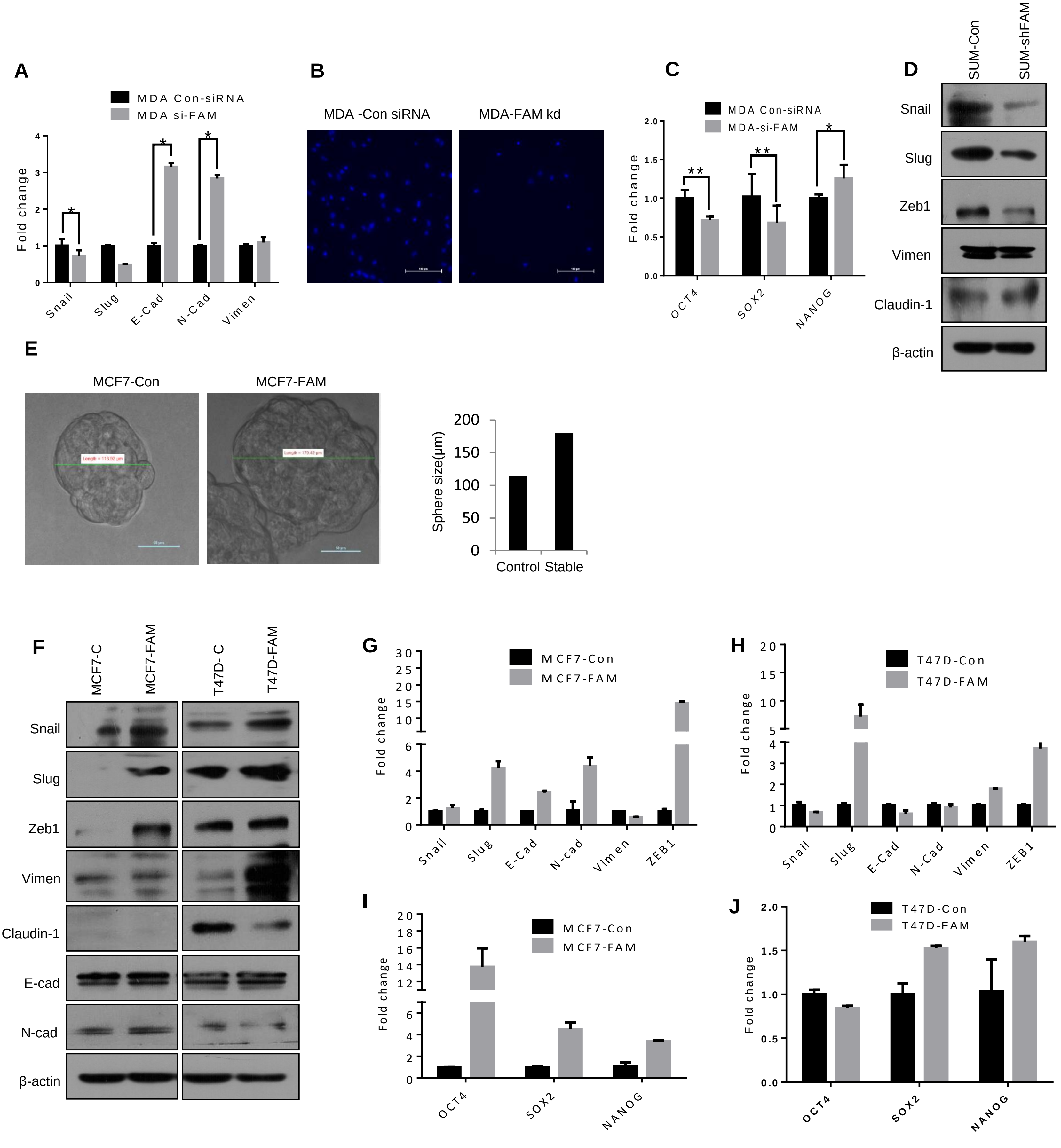

Supplementary Figure 3

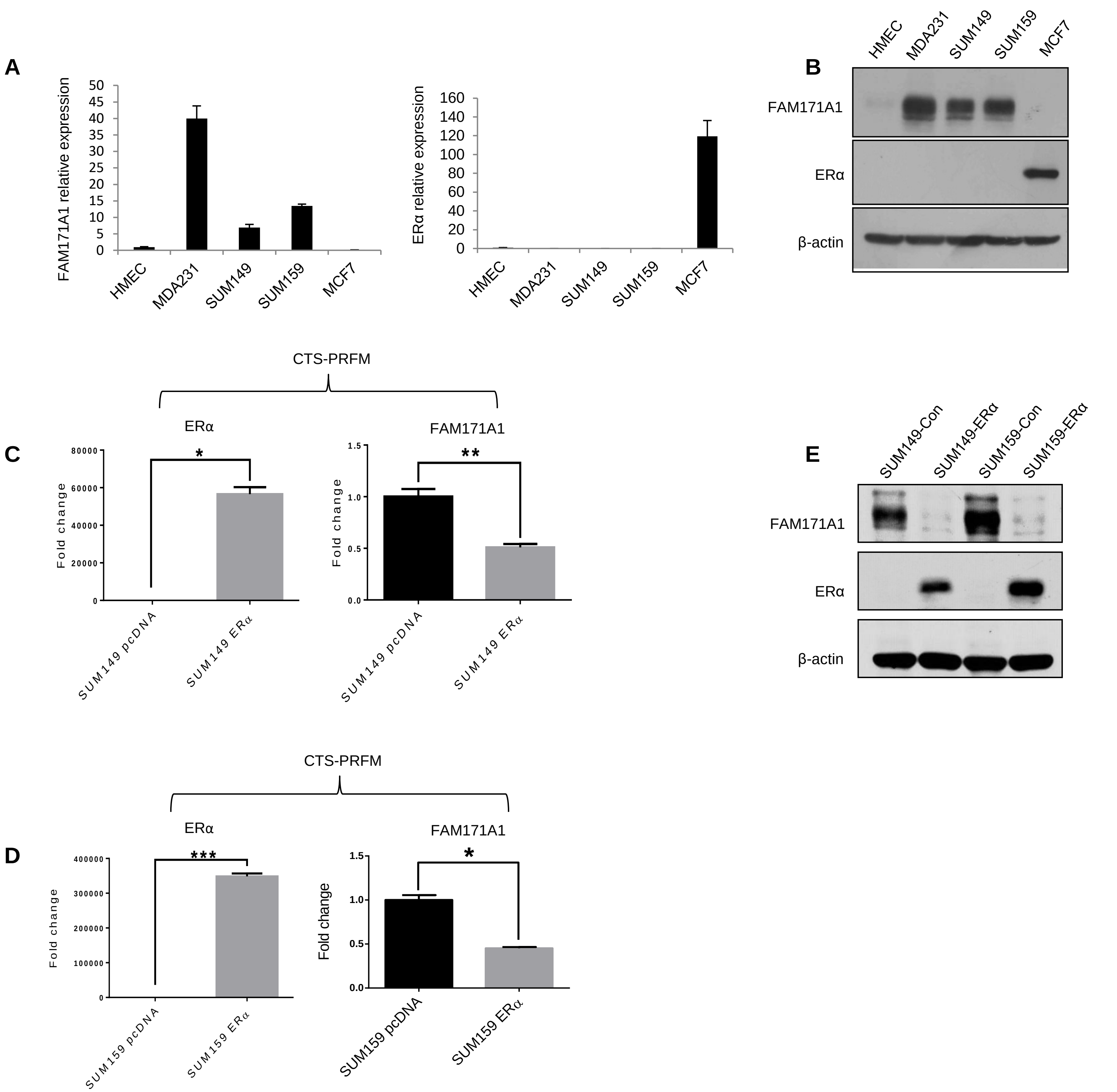

Supplementary Figure 4

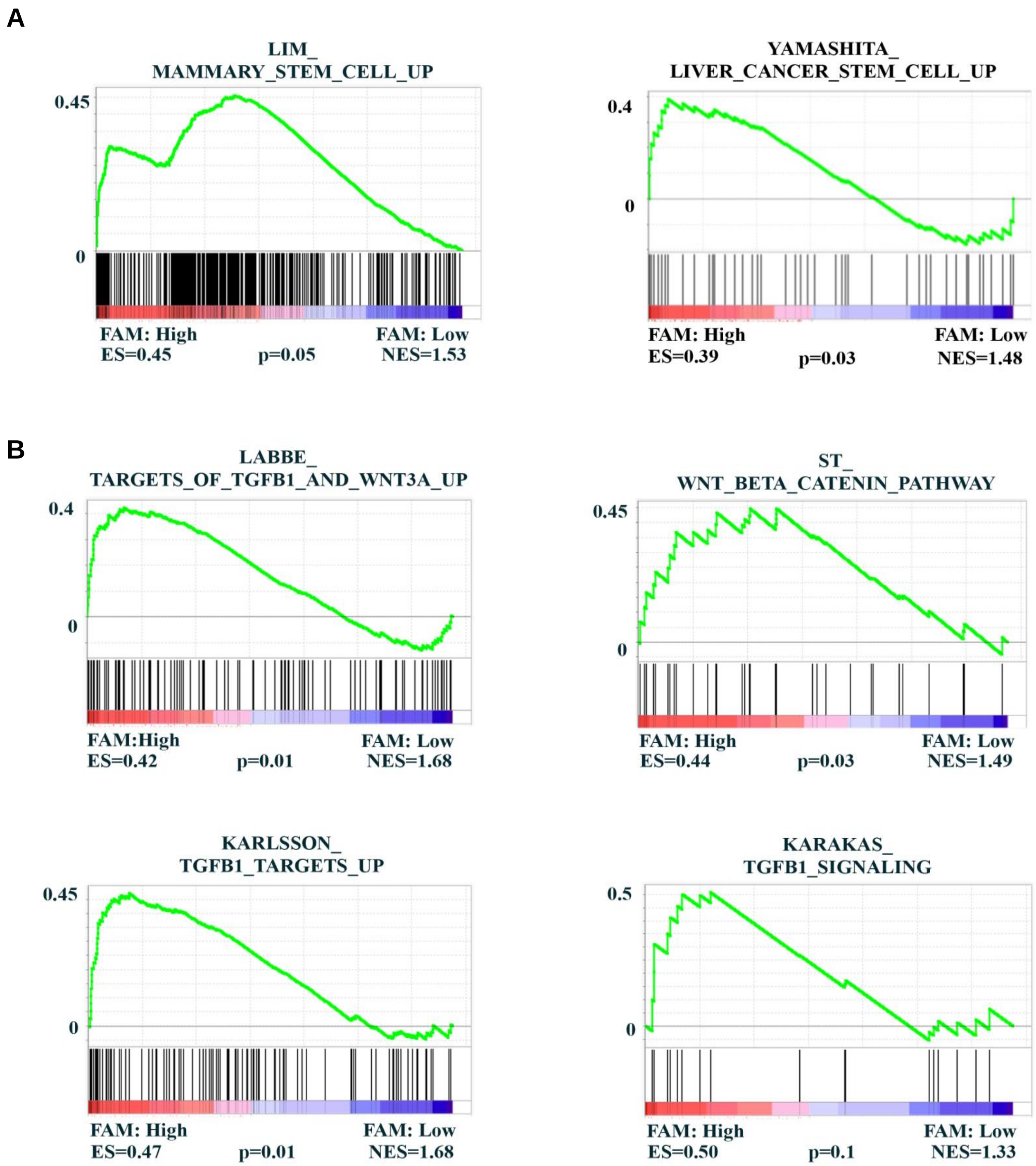

Supplementary Figure 5

SUPPLEMENTARY INFORMATION

Figure S1: (A) Expression of FAM171A1 mRNA in TNBC and non-TNBC cell lines and tumors from GSE36693 dataset. **(B)** Effect of tunicamycin, a glycosylase inhibitor, on the electrophoretic mobility of FAM171A1. **(C)** Kaplan-Meier survival curve analyses of FAM171A1 expression in breast cancer patients in van´t Veer (n=295) and BRCA-TCGA (n=962) datasets; data was stratified based on high (red) and low (green) FAM171A1 expression; log-rank test. **(D)** Immunoblot result showing silencing of FAM171A1 to see the effect on ERα in MDA-MB-231 cells. **(E, F)** qRT-PCR analysis of the effect of overexpression of ERα on FAM171A1 in MDA-MB-231 cells cultured in DMEM containing FBS (FBS-DMEM) as well as in phenol red free DMEM containing CTS (CTS-PRFM).

Figure S2: (A) Expression heat map of FAM171A1, ESR1 and PGR in NCI-60 Cell lines (NCI, Cancer Res. 2012) (n=60) and Cancer Cell Line Encyclopedia (Novartis/Broad, Nature 2012) (n=1020). **(B)** Heat map of FAM171A1, ESR1 and PGR in TCGA ; Breast Invasive Carcinoma TCGA Provisional (n=1105); Tyroid Carcinoma TCGA, Provisional (n=516); and Pancreatic Adenocarcinoma TCGA Provisional (n=186). **(C)** Scatter plot analysis of PGR and FAM171A1 mRNA expression in Breast Invasive Carcinoma dataset, TCGA Pan Atlas (n=1084) from cBioPortal; Pearson Correlation: -0.220; Spearman Correlation: -0.372. **(D**) Boxplots showing the mRNA expression of FAM171A1 in three Oncomine datasets stratified based on PGR-positive and negative status, TCGA (n=593), Curtis (n=2136), and Hatzis (n=508).

Figure S3: (A) qRT-PCR analysis of the MET markers in MDA-MB-231 cells upon FAM171A1 silencing. **(B)** Immunofluorescence of Hoest dye stained nuclie in MDA-MB-231 cells upon FAM171A1 silencing. Scale bar: 100µm. **(C)**. qRT-PCR analysis of the stem cell markers in MDA-MB-231 cells upon FAM171A1 silencing. **(D)** Western blot analysis of EMT markers in SUM149C and SUM149 sh-FAM171A1 stable clone. **(E)** Representative image of a

single sphere generated from MCF-7 cells stable clones expressing FAM171A1 as compared to MCF-7 vector control. Control MCF-7/pcDNA Sphere size=113.92µm; MCF-7/FAM171A1 stable clone Sphere size=179.42µm. Scale bar: 50µm, quantitation on the right side. **(F)** Western blot analysis of EMT markers in MCF-7/FAM171A1 and T47D/FAM171A1 stable clones and their respective controls, stable clones no. 2 were used each for MCF7 and T47D. **(G, H)** qRT-PCR analysis of EMT markers in MCF-7/FAM171A1 and T47D/FAM171A1 and respective control cells. **(I, J)** qRT-PCR analysis of the stem cell markers in MCF-7 cells and T47D cells upon FAM171A1 overexpression.

Figure S4: (A) qRT-PCR analysis of the FAM171A1 and ERα in normal human mammary epethelial (HMEC) cells when compared to MDA-MB-231, SUM149 and SUM159 (positive controls for FAM171A1 whereas negative controls for ERα) and MCF-7 (positive control for ERα whereas negative control for FAM171A1). **(B)** Western blot analysis showing the expression of FAM171A1 and ERα in HMEC cell line compared to TNBC and non-TNBC cell lines. **(C, D)** qRT-PCR analysis of the effect of overexpression of ERα on FAM171A1 in SUM149 and SUM159 cells cultured in CTS containing phenol-red free DMEM (CTS-PRFM). **(E)** Western blot analysis showing the effect of ectopic overexpression of ERα on FAM171A1 in SUM149 and SUM159 cells cultured in CTS containing phenol-red free DMEM (CTS-PRFM).

Figure S5: High expression of FAM171A1 is associated with activated Cancer Stem Cell (CSCs) gene signatures and associated TGF-β/Smad and Wnt/β-catenin signalling genes. **(A)** Gene set enrichment analysis (GSEA) showing positive correlation between high FAM171A1 expression and stem cell gene signatures (LIM_MAMMARY_STEM_CELL_UP; YAMASHITA_LIVER_CANCER_STEM_CELL_UP) in published breast cancer dataset GSE36693. **(B)** Gene set enrichment analysis (GSEA) plot showing the positive correlations between high expression of FAM171A1 and CSCs associated pathways- Wnt/β-catenin and TGF-β/Smad pathway gene signatures (KARAKAS_TGFB1_SIGNALING; KARLSSON_TGFB1_TARGETS_UP; LABBE_TARGETS_OF_TGFB1_AND_WNT3A_UP; ST_WNT_BETA_CATENIN_PATHWAY) in published GSE36693 data set.

Supplementary experimental procedures

Tunicamycin assay: MDA-MB-231 cells were seeded in 6-well plates and grown until about 70% confluency in culture medium containing 10% FBS. Cells were treated with 10 nM tunicamycin for 2 hrs, and cell lysates were resolved onto an 8% SDS-PAGE gels.
