# Supplementary material for: Estrogen receptor-α regulation of microRNA-590 targets FAM171A1—a modifier of breast cancer invasiveness
Source: Oncogenesis. 2019 Jan 9;8(1):5. doi: 10.1038/s41389-018-0113-z (PMC6328622; doi:10.1038/s41389-018-0113-z)
Supplement: Supplementary file 2 — REBUTTAL LETTER [file 41389_2018_113_MOESM2_ESM.docx]

Reviewer # 1

1) Abstract/Introduction. The author state that the ability to form mammospheres is directly proportional to the cancer aggressiveness? This statement should be referenced in the introduction section. (Major)

Response: Thank you for the comment. To support the noted positive correlation between mammospheres and aggressiveness, we have now corrected the statement in the abstract and provided a reference at the end in the text.

Page 2, lines 30-34: “Further, we found that the levels of *FAM171A1* correlate well with cancer cell aggressiveness as depletion or overexpression of *FAM171A1* confers reduced or increased ability of TNBC cells to form mammospheres, respectively , respectively in accordance with the previous report of increased mammosphere formation potential of metastatic cells ^13^”

Page 10, lines 210-220: “We found that *FAM171A1* overexpression in MCF-7 and T47D cells leads to increased mammospheres forming capability (Figure 5B, C, E, F and Supplementary Fig. 3E) as well as EMT markers such as Snail, Slug, Zeb1 and N-Cad in case of MCF-7/*FAM171A1* cells whereas Snail, Slug, Zeb1, Vimentin and Claudin-1 in case of T47D/*FAM171A1* stable cells (Supplementary Fig. 3F, 3G and 3H). These differences could be attributed to the differences in the cell line’s gene expressions. Ectopic expression of *FAM171A1* also increased stem cell markers such as Oct4, Sox2, and Nanog as observed in MCF-7/*FAM171A1* as well as T47D/*FAM171A1* stable clones (Supplementary Fig. 3I and 3J) as compared to the control cells. These results suggest that one of the roles of *FAM171A1* in breast cancer cells might be to support their stemness, and in-turn, aggressiveness^13^.”

2) Introduction. "In addition, FAM171A1 has been shown to interact with FAM171B....." Is not clear "in addition" to what, If these genes are important in BrCa or others tumors the authors should extend this sentence. (Minor)

Response: Thank you for the comment. We have now amended our mistake and removed “In addition” from the start of the line. Also we added references showing the implications of these genes in breast cancers and other cancers.

Page 3, line 52-54.“FAM171A1 has been shown to interact with FAM171B, PCDHGB1, TNFRSF17, TMEM, CTDSPL, NTRK1 ^11, 12, 14, 24^ many of them have been implicated in various cancers ^2, 6, 7, 19, 21, 28^ ”

3) Introduction. The description and discussion of Figure 1 panel F have to be performed in the "results" and "discussion" sections respectively, and not in the "introduction"(Minor)

Response: We thank reviewer for a nice review. We have now removed this statement from the Introduction section and mentioned in the Result section (page 5, lines 102-104) and Discussion sections (page 11, line 237-240)

Page 5, lines 103-105: “However, Kaplan-Meier survival analyses of miR590-5p show that high expression of miR590-5p may lead to better overall survival rates of breast cancer patients, highlighting its significance in breast cancer biology in physiologically relevant setting (Figure 1F)”

Page 11, line 237-241: “High expression of *FAM171A1* is associated with overall poor survival of the breast cancer patients in contrast to miR590-5p, whose elevated expression may lead to better overall survival rates as assessed by the KM plots suggesting an inverse correlation between clinical significance of these two genes in the survival of breast cancer patients.”

4) Figure 1. In the panel B, is not shown the relative expression of FAM171A1 in ZR75 cell line which is included in the panels A and C. In panel C, SKBR3 is considered a TNBC cell line to calculate the p value between TNBC and non-TNBC. Moreover, in the description of this panel in the result section authors fail to list also the MCF10A cell line which is a non-TNBC but shows low levels of ERαlpha mRNA expression. The significance could be compromised. (Major)

Response: We very much appreciate the comment in pointing this error on our part as we missed adding the names of cell lines in referred panels A and C. We have now moved the data from ZR-75 cells to Figure 1B. We also re-arranged the RT-PCR panel by grouping MCF-10A cells with TNBCs whereas separating SKBR3 from ER+ ve (MCF-7, T47D and ZR-75) and ER-ve (BT549, MDA-MB-231, SUM149, SUM159, Hs-578T, HCC1937 and MCF-10A); and p-values were also corrected in ER+ve and ER-ve cell lines (Figure 1B and 1C). We also re-developed the blot after washing any non-specific faint band earlier observed in MCF10A cells and replaced with Figure 1A.

5) Results. 8th row of page 6: "...FAM171A1 and ERα are mutually...." is wrong since there is a description of a "tendency towards mutual exclusivity". (Minor)

Response: We have now updated the heat map data from TCGA Pan Cancer Atlas. Both FAM171A1 and ERα exhibits mutual exclusivity with the significant p-value 0.002.

Page 6, lines 115-116: **“**We found that indeed, the expression of *FAM171A1* and *ESR1* (ERα) are mutually exclusive (Figure 2A, B).”

6) Results. 17th to 20th rows of page 6: The authors cannot address the ERα influences to FAM171A1 expression analyzing only one cell line. (Major)

Response: We agreed with the comment and showed the influence of ERα on FAM171A1 in another two more TNBC cell lines-SUM149, SUM159. In both of these cell lines, we observed a similar trend of results noticed earlier in MDA-MB 231 cell line (Figure 3D, E, and Supplementary Figure 4C, D and E). Accordingly, we have now updated the main text on Page 7, lines136-137 and added legends for Supplementary text S4

Page 7, lines 137-138: “Similar results were reproduced in SUM149 and SUM159 cell lines (TNBCs) (Supplementary Fig. 4C, D and E).”

CTS-PRFM

FAM171A1

ERα

CTS-PRFM

FAM171A1

ERα

7) Results. Can the authors explain why they start to study almost only the MDA-MB231 cell line? (Major)

Response: We thank for the comment. Among other TNBC cell lines used in this study, MDA-MB-231 cell line is the most widely studied and well established cell line used for both *in-vitro* and *in-vivo* TNBCs experiment, with good efficiency of transfection.

8) Results. 2nd to the 4th rows of page 8: Is not clear why the authors chose this microRNA to further studies. Only for the prediction in some (not described) predictive software. (Major)

Response: We have chosen to focus on miR590 not only on the basis of prediction but also on the basis of previous data. Please note that miR590 has been previously shown to be expressed at a high level in ER + cell line (MCF-7) as compared to ER – cell line (MDA-MB-231) (cited reference 17). However, using tools from MicroRNA.org, we have now recognized 3 seed sequences within a single 3’-UTR sequence of FAM171A1.

Similarly, we have observed other miRNAs too with considerable good score (hsa-miR-7-5p, hsa-miR-182-5p, hsa-miR-3613-3p, hsa-miR-4600, hsa-miR-6760-5p and hsa-miR-6807-3p) that bind to the 3’-UTR region of FAM171A1 but did not select them for the further studies as we were unable to find literature showing their differential expression between ER+ vs ER-ve cell lines– as was the case with miR590-5p. Prediction database’s name used here for miR590-5p is “microrna.org” and now mentioned in the manuscript.

Page 8, lines 157-159: **“We identified miR590-5p as a candidate gene with three seed sequences within the single 3’-UTR sequence of *FAM171A1* using “microrna.org” database (Figure 3B).”**

Figure 3. In the panel C, the p-values were calculated excluding MCF10A and putting the SKBR3 in the TNBC cancer cell lines group. This cells are ERα negative (Figure 1A) but HER2 positive. (Major)

Response: We thank reviewer for identifying this error. We have now arranged these RT-PCR data on the basis of ER +ve vs ER –ve status keeping MCF10A under TNBC and excluding SKBR3 separately. RT-PCR result re-arranged, p-values corrected, (Figure 1B & 1C).

9) Figure 3. The IP described in panel F is not convincing. The Inputs of the negative DNA regions are too low in intensity to demonstrate the real negativity of the experiment. (Major)

Response: We thank our expert reviewer for identifying these concerns about the clarity of this image. We have re-performed the ChIP-PCR experiment and replaced the previous figure with the updated revised one (Figure 3F).

10) 1st and 2nd rows of page 9: How the authors explain the results described in figure 3I? In the rationale of figure 3, it seems that the hypothesis consisting of the direct target of the miRNA to the 3' UTR of the gene. However, the experiments do not prove this direct targeting. (Major)

Response: In Figure 3I we have shown that transfecting the cells with a combination of mir590-5p-pRIP vector and 3’-UTR-pSciCheck2 vector leads to a repression of FAM171A1 transcripts as evident by the noted reduction in the luciferase assay when compared to transfecting the cells with a combination of pRIP plasmid transfected with 3’-UTR-pSciCheck2. Hence, we show that mir590-5p negatively regulates FAM171A1 expression via binding to its 3’-UTR region.

Reviewer #2

1) By in silico analysis, the authors should also investigate if FAM171A1 levels in breast cancer are also dependent by amplification of FAM171A1 locus: is the FAM171A1 mRNA expression significantly associated with the presence of FAM171A1 copy number variation?

Response: We hanks for the suggestion. Accordingly, we have now explored the association between the mRNA expression and copy number variation of FAM171A1. We analyzed the following databases: Breast Invasive carcinoma (TCGA, Cell 2015), Breast Cancer (METABRIC Nature 2012, Nature Communication, 2016), Breast Invasive Carcinoma (TCGA, Pan Cancer Atlas, 2018), Breast Invasive Carcinoma (TCGA, Pan Cancer Atlas, 2018). It appears that FAM171A1 mRNA expression shows only moderate positive correlation with its copy number variation (Pearson: 0.44, Spearman: 0.25) (see below)

2) Is miR590-5p able to suppress the transcription of FAM171A1 when the gene is mutated? How frequent is the mutation of FAM171A1 in breast cancers? How relevant is the FAM171A1 gene mutation/amplification for the breast cancer disease? These questions need also to be addressed

Response: Thank you for these valuable comments. In general, miRNA doesn’t bind to the coding region of FAM171A1 instead it binds to the 3’UTR (untranslated region). Hence, even if gene is mutated within exon region, it is expected to code for an altered no protein. However, if there are mutations in the seed sequences for miR590-5p binding sites, we would expect no regulatory control of miR590-5p over FAM171A1 expression. Please note that in the present study, we did not focus on studying such mutations as we believed that this could be best developed as a standalone future study. Regarding the second comment, we have extensively evaluated potential mutation frequency of FAM171A1 within various available databases and found very low mutational frequency in the range of 0.6-1.1%

Breast Invasive Carcinoma (TCGA, Pan Cancer Atlas n=993)-1.1%

Breast Invasive Carcinoma (TCGA, Nature 2012) - 1.1%

Mutational profiles of metastatic breast cancer (France, 2016) -0.9%

Metastatic Breast Cancer Project (Provisional, April 2018) - 0.6%

Regarding the third valuable comment, we analyzed the correlation between the mutation vs survivability of the breast cancer patients. Mutation/alteration in FAM171A1 leads to better overall survival as well as Disease/Progression-free survival as observed in TCGA Breast Invasive Carcinoma datasets. This suggests that FAM171A1 might be an oncogene, possibly contributing to the noted poor survival of the breast cancer patients.

3) The authors showed that FAM171A1 was upregulated, at both protein and mRNA level, in the TNBC cell lines compared with the non-TNBC cell lines. But, how is the expression of FAM171A1 in the normal/immortalized mammary epithelial cells?

Response: We really appreciate the valuable and constructive comment. Please note that we have analyzed the endogenous expression levels of FAM171A1 in HMEC cell line together with TNBC and non-TNBC cell lines as positive and negative controls, respectively. WB & qRT-PCR result are attached in supplementary figure (Supplementary 4A, B). Accordingly, we have now updated the main text, page 5, line 96, and added an appropriate legend for Supplementary Figure S4

Page 5, line 97: “……………as well as normal mammary epithelial cell line, HMEC (Figure 1A, B, C, Supplementary 4A, B).”

|  | FAM171A1 relative expression |
| --- | --- |
| HMEC | 1.00168 |
| MDA231 | 39.975 |
| SUM149 | 6.93527 |
| SUM159 | 13.4671 |
| MCF7 | 0.128476 |

|  | ERα relative expression |
| --- | --- |
| HMEC | 1.00112 |
| MDA231 | 0.0187439 |
| SUM149 | 0.0301523 |
| SUM159 | 0.0156953 |
| MCF7 | 119.445 |

4) The authors should investigate in vitro (e.g. counting the number of apoptotic cells by FACS analysis) if FAM171A1 may contribute to therapeutic resistance of TNBC cell lines: does FAM171A1 silencing enhance the sensitivity of TNBC cells to chemotherapeutic drugs?

Response: We thank reviewer for raising these interesting possibilities. In this context, while analyzing previously published microarray datasets, we did notice some clues, suggesting that FAM171A1 might be involved in Lapatinib and doxorubicin resistance (reference 5 & 16). However, in the present study, we did not focus on studying this aspect of FAM171A1 biology as we believed that this could be best developed as a standalone future study inclusive of underlying molecular details. We are sorry for not expending into the suggested peripheral (yet, important) goal of the current study.

5) To further suggest the involvement of FAM171A1 in regulating cancer stem cell-like traits, the authors should investigate, by in silico analysis (e.g. Gene Set Enrichment Analysis), if there is an overlap between high expression profiles of FAM171A1 and stem cell signatures

Response: We thank the reviewer for the suggestion. Accordingly, we have now performed GSEA analysis for both stem cell signatures as well as pathways associated with stem cells. We updated the text in the manuscript at page 10, lines 199-206.

Page 10, lines 200-207: “Further, to evaluate its association with stemness, we utilized Gene Set Enrichment Analysis (GSEA) of GSE36693 dataset in order to see any possible overlap between profiles with high expression of *FAM171A1* and stem cell gene signatures. Analysis revealed that high expression of *FAM171A1* correlates with upregulated stem cell gene signatures and therefore may be involved in the regulation of cancer stem cell functions (Supplementary Fig. 5A). Additionally we also observed activated gene signatures for TGFβ/Smad and Wnt/β-catenin pathways ^20, 27^ suggesting these stem cell associated pathways could possibly be linked with *FAM171A1* (Supplementary Fig. 5B).”

Results are attached in the supplementary (Supplementary 5A, B) and added a respective legend for Supplementary Figure S5.

**A**

**B**

6) Since the BT549 is the TNBC cell line with the highest level of expression of FAM171A1 (as shown in Figure 1B), this breast cancer cell line should be used by authors as the primary TNBC cell line to support their in vitro results about the significance of FAM171A1 expression levels in TNBC cancers

Response: We appreciate reviewer’s comment and would like to answer for the same. In our experience, as compared to MDA-MB-231 cells, we had lots of difficulties in growing the BT549 cells as these cells grow in gummy appearance making them unsuitable for transfection studies or creating stable cell lines. Also MDA-MB 231 cells are widely studied and are widely preferred model for TNBC studies.

7) The discussion section is totally missing. The authors must to critically interpret their results, and compare/discuss what they have obtained with the current research findings on the argument

Response: We very much appreciate the reviewer for identifying this issue. Now, we have added a brief Discussion section and updated the manuscript according to the revised manuscript.

Minor issues:

8) The resolution of the Kaplan-Meier plots needs to be improved: actually the plots show a too low resolution

Response: Thanks for the critical evaluation. We have retrieved these Kaplan-Meier plots from the online databases hence the resolution appear as uploaded in the databases. However we have agreed to your comment and have increased the resolution of Kaplan-Meier plots to the possible extent. Also we increased the size of the plots and typed high and low over/below the respective lines for better visualization of the line colors.

9) Figure 1E and Supplementary Figure 1C: the authors said that "increased expression of FAM171A1 and its levels correlate well with an overall poor survival of TNBC patients as compared to non-TNBC patients" ("page 5" of the manuscript). Which of the reported plots in Figure 1E and Supplementary Figure 1C are referred to TNBC patients, and which one are referred to non-TNBC patients?

Response: There might be some confusion here as we might have not clearly stated these facts. Please note that we did mention that “high expression of *FAM171A1* and its levels correlate well with an overall poor survival of TNBC patients as compared to non-TNBC patients (Figure 1E and Supplementary Fig. 1C)”. Here, both Figure 1E as well as Supplementary Figure 1C represents the same conclusion, i.e. elevated expression leads to the overall poor survival. We have kept two main KM survival curves (Figure 1E) whereas another two KM survival curves as supporting data in the Supplementary Fig. 1C (High expression correlates to TNBC patients and low expression correlates to non-TNBC patients here everywhere in plots)

10) Figure 1E: the figure legend reports that the plots are stratified "based on high (red) and low (blue)" FAM171A1 levels, but the right (Kaplan-Meier) plot actually report "Green" and "Red" lines

Response: Authors thank the reviewers for their critical review and identifying these errors. We have now corrected the main figure legend (Figure 1E), page 25, lines 547-549.

Page 24, lines 516-518: “datasets stratified based on high (red) and low (blue) in case of GSE3143 dataset and high (red) and low (green) in case of Breast Invasive Carcinoma TCGA”

11) Figure 1F: the figure legend reports that the plots are stratified "based on high (red) and low (blue)" hsa-miR590-5p levels, but both plots actually show "Black" and "Red" lines

Response: We thank the reviewers for their critical evaluation. We now have corrected the main figure legend (Figure 1F).

Page 24, line 520:“datasets stratified based on high (black) and low (red)”

12) Supplementary Figure 1 C: the color lines of the K-M plots are "Green" and "Red", and not "Red" and "Blue" lines are present as, instead, reported in the supplementary information legend

Response: We thank you for the comment. We have corrected the supplementary figure legend for Supplementary figure 1C legend, line 5.

Figure S1, Line 5: “data was stratified based on high (red) and low (green) FAM171A1 expression”

13) Figure 2D, Figure 2E, Supplementary Figure 1E, and Supplementary Figure 1F: each plot should be provided with a header to show clearly under which conditions the Western Blots/qRT-PCR analyses have been conducted (i.e. “DMEM with FBS” and “Phenol-red free DMEM with CTS”)

Response: We appreciate the comment. Accordingly, we have added the headers to the mentioned figures (Figure 2D, Figure 2E, Supplementary Figure 1E and Supplementary Figure 1F).

14) Supplementary Figure 3A and Figure 3C: the p-values of these plots are missing

Response: We thank the reviewer for identifying the mistake. We have added the p-values in the revised figure, (Supplementary Figure 3A and Supplementary Figure 3C)

15) Supplementary Figure 3: how about the qRT-PCR data of the stem cell markers (i.e. Oct4, Sox2, and Nanog) in T47D cells upon FAM171A1 silencing?

Response: We thank the reviewer for the constructive comment. We have added a qRT-PCR data for stem cell markers in T47D cell linetoo.

Page 10, line 217: “as well as T47D/*FAM171A1* stable clones (Supplementary Fig. 3I and 3J)”

16) Typos errors are present throughout the manuscript

Response: We thank you for the comment towards improving the manuscript quality. We have improvised the manuscript, corrected MDAMB231 to MDA-MB-231 and MCF7 to MCF-7 throughout in the manuscript, and made all other recommended corrections.

17) The gene names must to be italicized

Response: We have corrected the gene names in italicized format whereas protein names are kept unchanged as such.
